# Trehalose inhibits H_2_O_2_-induced autophagic death in dopaminergic SH-SY5Y cells via mitigation of ROS-dependent endoplasmic reticulum stress and AMPK activation: Erratum

**DOI:** 10.7150/ijms.89278

**Published:** 2023-09-08

**Authors:** Zhijie Gao, Helei Wang, Bo Zhang, Xuemei Wu, Yanfeng Zhang, Pengfei Ge, Guangfan Chi, Jianmin Liang

**Affiliations:** 1 Department of Neurosurgery, First hospital of Jilin University, Changchun 130021, China.; 2 Department of Gastrointestinal Surgery, First hospital of Jilin University, Changchun 130021, China.; 3 Department of Pediatric Neurology, First hospital of Jilin University, Changchun 130021, China.; 4 Research center of neuroscience, First hospital of Jilin University, Changchun 130021, China.; 5 Key Laboratory of Pathobiology, Ministry of Education, Jilin University, Changchun 130021, China.

The authors regret that they divided the blots from the same groups of samples into two images (Fig. 4 C and 4D) in consideration of layout neatness but used the same β-actin. Although Fig. 4 C and D were both from the same group of samples, it is not properly to use the same β-actin in Fig. 4 C and 4D. Similar blot division and usage of the same β-actin could also be found in Fig 5B and 5C. The authors would like to merge figure Fig. 4C and D into one image, and merge figure Fig.5B and 5C into one image. Thus, they make this corrigendum.

## Figures and Tables

**Figure 4:**
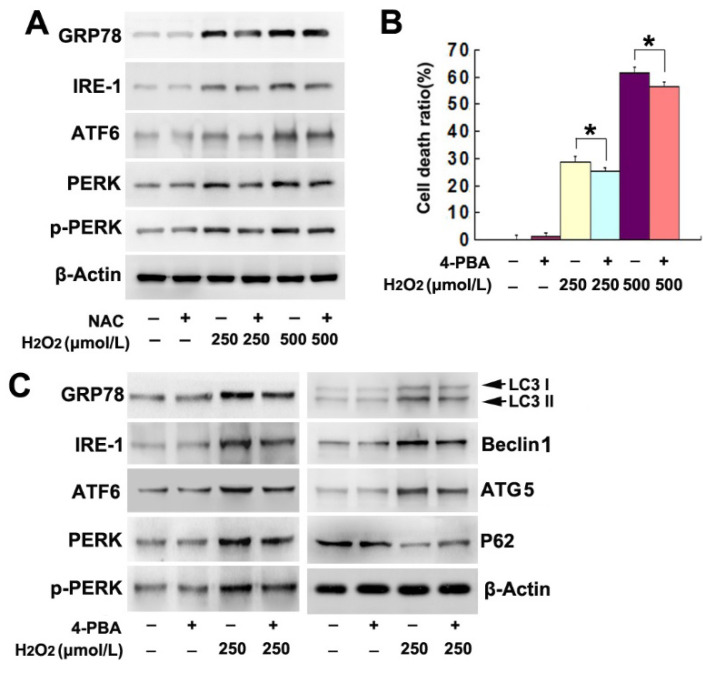
Corrected figure.

**Figure 5:**
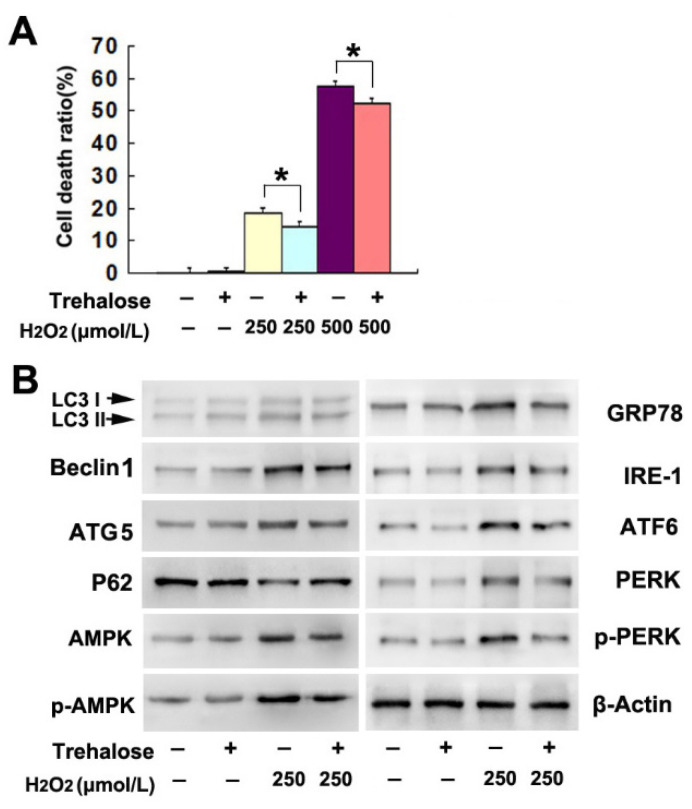
Corrected figure.

